# Variations in Fruit Ploidy Level and Cell Size between Small- and Large-Fruited Olive Cultivars during Fruit Ontogeny

**DOI:** 10.3390/plants13070990

**Published:** 2024-03-29

**Authors:** Maria C. Camarero, Beatriz Briegas, Jorge Corbacho, Juana Labrador, Ángel-Carlos Román, Antía Verde, Mercedes Gallardo, Maria C. Gomez-Jimenez

**Affiliations:** 1 Laboratory of Plant Physiology, Universidad de Extremadura, Avda de Elvas s/n, 06006 Badajoz, Spain; 2 Department of Molecular Biology, Biochemistry and Genetics, Universidad de Extremadura, Avda de Elvas s/n, 06006 Badajoz, Spain; 3 Laboratory of Plant Physiology, Universidad de Vigo, Campus Lagoas-Marcosende s/n, 36310 Vigo, Spain

**Keywords:** cell division, cell expansion, flow cytometry, fruit development, fruit size, olive

## Abstract

Olive (*Olea europaea* L.) is one of the major oil fruit tree crops worldwide. However, the mechanisms underlying olive fruit growth remain poorly understood. Here, we examine questions regarding the interaction of endoreduplication, cell division, and cell expansion with olive fruit growth in relation to the final fruit size by measuring fruit diameter, pericarp thickness, cell area, and ploidy level during fruit ontogeny in three olive cultivars with different fruit sizes. The results demonstrate that differences in the fruit size are related to the maximum growth rate between olive cultivars during early fruit growth, about 50 days post-anthesis (DPA). Differences in fruit weight between olive cultivars were found from 35 DPA, while the distinctive fruit shape became detectable from 21 DPA, even though the increase in pericarp thickness became detectable from 7 DPA in the three cultivars. During early fruit growth, intense mitotic activity appeared during the first 21 DPA in the fruit, whereas the highest cell expansion rates occurred from 28 to 42 DPA during this phase, suggesting that olive fruit cell number is determined from 28 DPA in the three cultivars. Moreover, olive fruit of the large-fruited cultivars was enlarged due to relatively higher cell division and expansion rates compared with the small-fruited cultivar. The ploidy level of olive fruit pericarp between early and late growth was different, but similar among olive cultivars, revealing that ploidy levels are not associated with cell size, in terms of different 8C levels during olive fruit growth. In the three olive cultivars, the maximum endoreduplication level (8C) occurred just before strong cell expansion during early fruit growth in fruit pericarp, whereas the cell expansion during late fruit growth occurred without preceding endoreduplication. We conclude that the basis for fruit size differences between olive cultivars is determined mainly by different cell division and expansion rates during the early fruit growth phase. These data provide new findings on the contribution of fruit ploidy and cell size to fruit size in olive and ultimately on the control of olive fruit development.

## 1. Introduction

Fleshy fruit undergo a precise orchestration of steps, including fruit set, growth, maturation, and ripening, by integrating endogenous signals and various environmental cues [1,2,3,4]. During fruit growth, the final fruit size is a complex trait that results from strict spatiotemporal control and coordination of overlapping and interconnected cellular events, cell division, and cell expansion, beginning at different times and having varying rates as well as duration [5,6]. Notable progress has been made in recent years in elucidating the cellular and molecular mechanisms controlling fruit growth [3,4,5,6,7]. Regarding the cell level, previous studies have linked endoreduplication with cell growth, especially in tomato fruit, in which high levels of endopolyploidy occur during fruit growth [8,9,10,11]. Moreover, it has been shown that cell division, endoreduplication, and cell expansion are triggered simultaneously in specific cell layers by the same signals transmitted from fertilization, which contribute to the fastest relative tomato fruit growth soon after fruit set [12]. Similarly, in several fruit species, correlations between the ploidy level and cell size have also been reported [13,14,15,16]. However, in olive (*Olea europaea* L.) fruit, no studies available have investigated the role of endoreduplication in fruit size control.

In olive, fruit size is a quality trait of interest mainly for table olive cultivars [17,18]. The extensive genetic resources available for olive are illustrated by a wide variability of many characters of olive fruit [19,20,21]. Recent studies have identified, using genome-wide association and RNA-sequencing analyses, candidate genes associated with key morphological traits of olive fruit, including fruit size and weight [20,21]. In this context, we identified novel candidate genes previously unconnected with cell division and cell expansion phases during early development of olive fruit [22]. In terms of growth, the analysis of the olive fruit histology using qualitative methods has shown that cultivar-based fruit size was related directly to cell number and was established soon after anthesis by cell division rate in olive cultivars [23,24,25,26,27]. Likewise, olive fruit growth and sink strength are related to cell number, not to tissue size [28,29]. However, to date, the ploidy level during olive fruit development is still poorly investigated [22,30]. Recently, we determined the duration and rate of the fruit cell division phase in olive using flow cytometry [22]. In fact, we provided the first detailed quantitative analysis at the ploidy level during the early development of olive fruit, showing that the maximum relative rate of cell division was found at 14 days after anthesis, and a significant proportion of endoreduplicated cells of up to 8C was detected [22].

Based on our previous data, in the present study, we consider the variability of olive fruit size in order to address the question of its dependence on fruit ploidy and cell size during fruit ontogeny. For this, we made a quantitatively comparative analysis of fruit development in three olive cultivars that differed in the final fruit size. This included cytology and ploidy analyses associated with fruit development in an olive small-fruited cultivar, ‘Arbequina’, and two large-fruited cultivars, ‘Picual’ and ‘Manzanilla Sevillana’. Specifically, we examined mitotic activity, using flow-cytometric analysis, and mesocarp cell area of the developing olive fruits to determine whether cell division and/or cell expansion might be reduced in the small fruit, or conversely increased in the case of large fruit. In addition, to demonstrate the role of endoreduplication in olive fruit growth control, we investigated the cell ploidy profile during early and late fruit development in pericarps of the three olive cultivars.

## 2. Results

### 2.1. Morphological Changes during Fruit Development in Olive Cultivars

**Figure 1:**
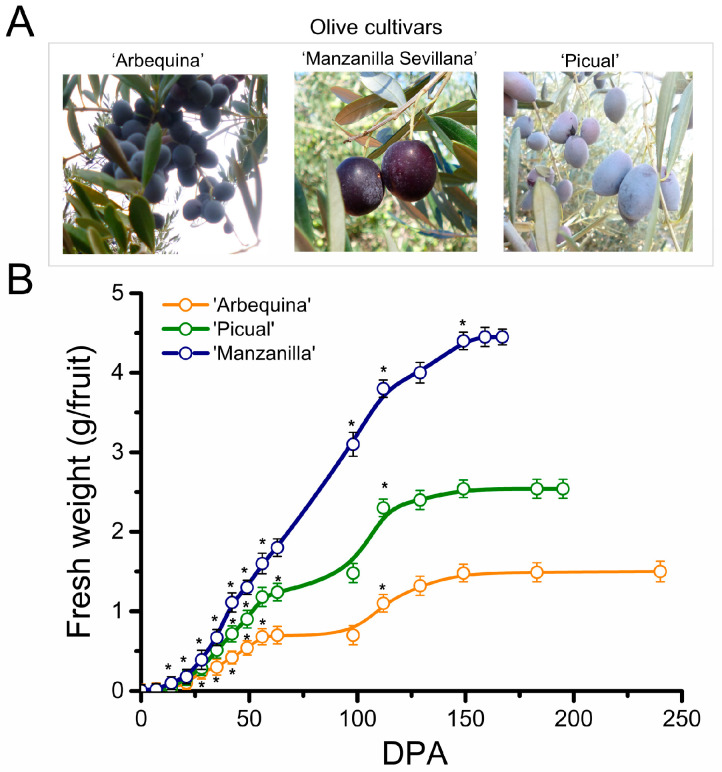
Fruit development of ‘Arbequina’, ‘Manzanilla Sevillana’, and ‘Picual’ olive cultivars with contrasting fruit size and shape. (**A**) Images of olive fruit morphology at the fully ripe stage in ‘Arbequina’, ‘Manzanilla Sevillana’, and ‘Picual’ cultivars at 260, 169, and 189 DPA, respectively. (**B**) Changes in fresh weight (FW) (g fruit^−1^) of olive fruit during fruit growth and ripening in the three cultivars: ‘Arbequina’ with small and round fruit, ‘Manzanilla Sevillana’ with large and round fruit, and ‘Picual’ with large and elongated fruit. Each point is the average of 20 fruits. The values were estimated as means ± SD. Asterisks indicate statistically significant changes with respect to the preceding point according to Tukey’s test (*p* < 0.05). The differences observed among cultivars are presented in Table S1. DPA: days post-anthesis.

The characteristics of olive developing fruits were compared in the three olive cultivars, ‘Arbequina’, ‘Manzanilla Sevillana’, and ‘Picual’, which were chosen because of the different fruit sizes (Figure 1A). The variations in the fruit weight between olive cultivars, in which the mean fruit weights at fully ripe stage ranged from 1.5 to 4.5 g, were discernible from 35 days post-anthesis (DPA) under the same growing conditions (Figure 1B, Table S1). The fruit weight difference among the three cultivars gradually increased as a result of growth after 35 DPA until fruit ripening (Figure 1B, Table S1). The endocarp lignification of olive fruit is considered to undergo growth arrest around 50 days after pollination and fertilization [22]. Fruit ripening began at 140–150 DPA, as indicated by color change of the olive fruit, and continued to full ripeness. Although the three cultivars initially gained fruit weight rapidly, the small-fruited cultivar ‘Arbequina’ required some 240 DPA to reach complete fruit ripeness, as indicated by the fully black color of the olive fruit, whereas the large-fruited cultivars, ‘Picual’ and ‘Manzanilla Sevillana’, required about 195 and 169 DPA, respectively (Figure 1B).

In the present study, the results were recorded in two independent experiments, the first from anthesis to 50 DPA (early fruit development) and the second from 50 DPA to fully ripe stage (late fruit development).

### 2.2. Early Fruit Development in Olive Cultivars

**Figure 2:**
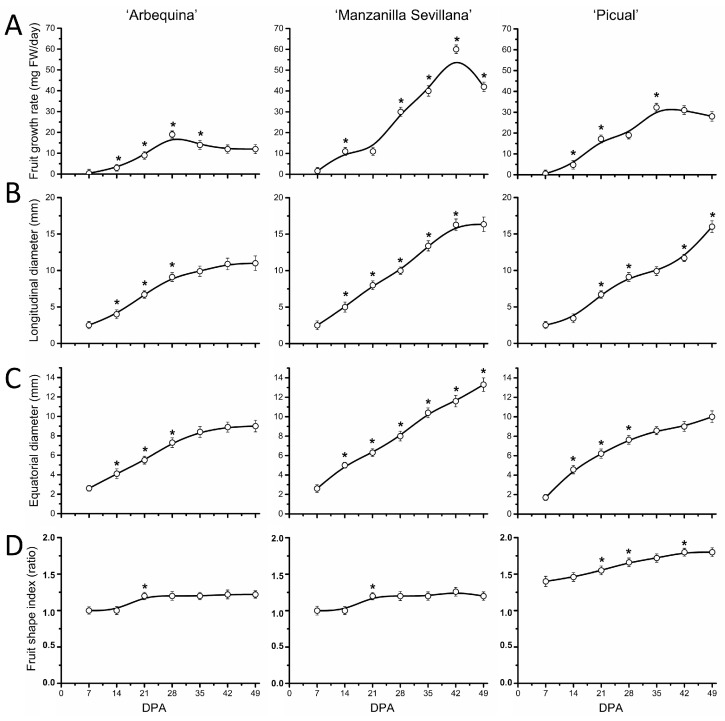
Morphological changes of olive fruit of ‘Arbequina’, ‘Manzanilla Sevillana’, and ‘Picual’ cultivars during early fruit development. (**A**) Changes in growth rate (mg FW day^−1^), (**B**) longitudinal diameter (mm), (**C**) transverse diameter (mm), and (**D**) fruit shape index of developing olive fruit at 0, 7, 14, 21, 28, 35, 42, and 49 DPA from the three olive cultivars. Fruit shape index is length-to-width ratio of the fruit. Each point is the average of 5 fruits. The values were estimated as means ± SD. Asterisks indicate statistically significant changes with respect to the preceding point according to Tukey’s test (*p* < 0.05). The differences observed among cultivars are presented in Table S1.

First, to appreciate the extent of this growth arrest on endocarp lignification of olive fruit, we measured various growth-related variables in the ovary and fruit of the three olive cultivars, from anthesis (0 DPA) up to 49 DPA, as described elsewhere [22]. Even though the fruit weight increases were largest between 21 and 42 DPA in all olive cultivars, their growth rates reached maximum at 28 DPA in ‘Arbequina’, at 35 in ‘Picual’, and at 42 in ‘Manzanilla Sevillana’ (Figure 2A). In the small-fruited cultivar ‘Arbequina’, the olive fruit growth rate slowed after 28 DPA, but accelerated in large-fruited cultivars (up to 35 and 42 DPA in ‘Picual’ and ‘Manzanilla Sevillana’, respectively). Values for this variable then decreased during the endocarp lignification in all three olive cultivars, indicating the end of the early vigorous growth period of the olive fruit. In addition, the maximum growth rate was slower in the small-fruited cultivar, ‘Arbequina’, than in the large-fruited cultivars, ‘Manzanilla Sevillana’ and ‘Picual’ (Figure 2A, Table S1). That is, ‘Arbequina’ fruit weight was augmented 64-fold from anthesis to 42 DPA, whereas in both ‘Picual’ and ‘Manzanilla Sevillana’, the values increased 128-fold, doubling the growth of ‘Arbequina’ from anthesis to 42 DPA (Figure 1B). Thus, under the same growing conditions, the three olive cultivars varied in the duration of early fruit growth and in the maximum growth rate.

Regarding fruit shape, it bears mentioning that in ‘Arbequina’ and ‘Manzanilla Sevillana’ cultivars, fruit length and width showed similar trends during this phase, resulting in a rounder shape of these cultivars; however, in ‘Picual’, with elongated fruit, the growth in length was the most rapid followed by width starting from 14 DPA (Figure 2B,C), resulting in an increase in the fruit shape index after 21 DPA (Figure 2D).

**Figure 3:**
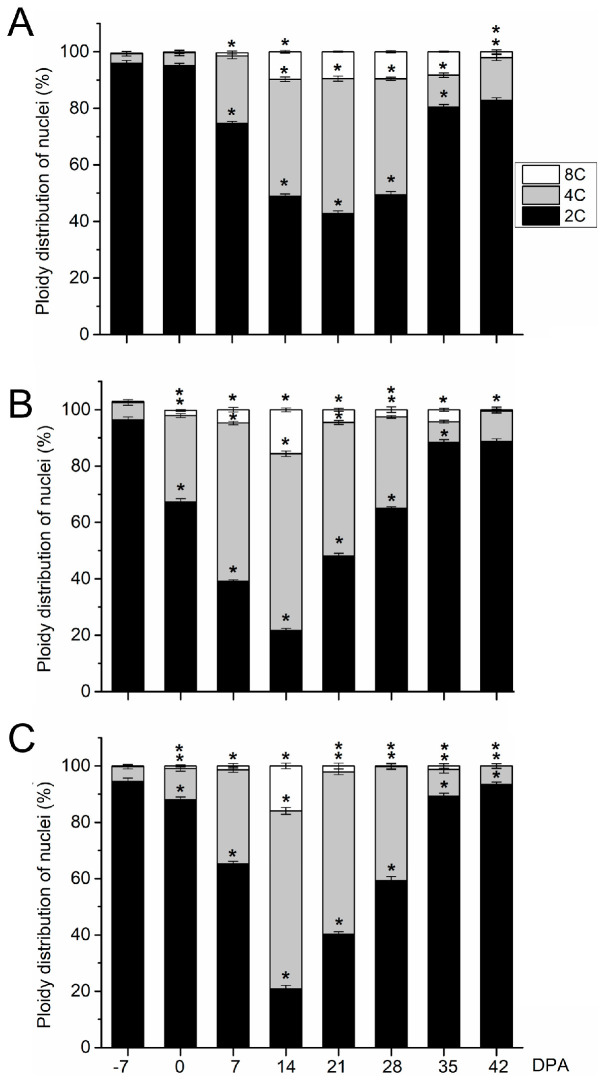
Nuclear ploidy levels from olive fruits during early development in (**A**) the ‘Arbequina’, (**B**) ‘Manzanilla Sevillana’, and (**C**) ‘Picual’ olive cultivars. The percentage of nuclei in 2C, 4C, and 8C are shown from 0 to 42 DPA in the developing fruits. Each point is the average of four samples. Asterisks denote significant differences based on Tukey’s test (*p* < 0.05) from the preceding point, and bars are ± SD. The differences observed among cultivars were presented in Table S1.

Next, flow cytometry to detect cell division activity and endoreduplication was performed on whole fruits of the three olive cultivars from 0 to 49 DPA (Figure 3). In olive fruit of the small-fruited cultivar, the maximal proportion of 4C cells (47.8%), which is an indirect estimate for cell division [31,32], was reached at 21 DPA (Figure 3). High levels of cell division were found afterwards at 21 DPA, but the proportion of 4C cells was lower in ‘Arbequina’ (47.8% for the maximum level) than in ‘Manzanilla’ and ‘Picual’ fruits (62.7% and 63.1% for the maximum level, respectively; Figure 3, Table S1). The slower division rate of ‘Arbequina’ fruit suggests a lower final cell number compared with ‘Manzanilla’ and ‘Picual’ fruits at this stage. Notably, at 35 DPA, the proportion of 2C cells increased while the proportion of 4C cells decreased remarkably in ‘Arbequina’ fruits (Figure 3), indicating that cell division had almost completely stopped. The 8C cells represented 9.7% and 8.2% of the cells in ‘Arbequina’ fruits at 14 and 35 DPA, respectively, suggesting that ‘Arbequina’ fruits undergo endoreduplication from 14 to 35 DPA.

In large-fruited cultivars, ‘Manzanilla Sevillana’ and ‘Picual’, the analyses of cell division activity showed similar trends during early olive fruit development. From anthesis (0 DPA) to 14 DPA, the proportion of 4C cells increased with time, while the proportion of 2C cells decreased in olive fruits (Figure 3). At 14 DPA, 4C cells represented the highest proportion of cells in the olive fruits (62.7% and 63.1% in ‘Manzanilla’ and ‘Picual’, respectively), indicating intensive cell division. By contrast, at 28 DPA, cell division was not activated in fruits based on the increased ratio of 2C to 4C DNA levels relative to that at 21 DPA (Figure 3). From 28 to 42 DPA, most of the nuclei were 2C (88.7% and 93.5% of total nuclei of fruits at 42 DPA in ‘Manzanilla’ and ‘Picual’ cultivars, respectively), while only low proportions of 4C and 8C nuclei were detected in these fruits (Figure 3). Thus, the results showed that cell division was triggered by pollination in the ‘Manzanilla Sevillana’ and ‘Picual’ olive fruits from 0 to 21 DPA. In addition, the proportion of 8C cells at 14 DPA increased in comparison to those in fruits at 7 DPA (15.6% and 15.8% of total nuclei at 14 DPA in ‘Manzanilla’ and ‘Picual’, respectively; Figure 3), showing that the 8C proportion of both ‘Manzanilla’ and ‘Picual’ fruits at 14 DPA were higher in comparison to ‘Arbequina’ fruit at 14 DPA (Table S1). However, the proportion of 8C cells in ‘Arbequina’ fruits remained up to 35 DPA, while this proportion decreased in both ‘Manzanilla’ and ‘Picual’ fruits (Figure 3).

**Figure 4:**
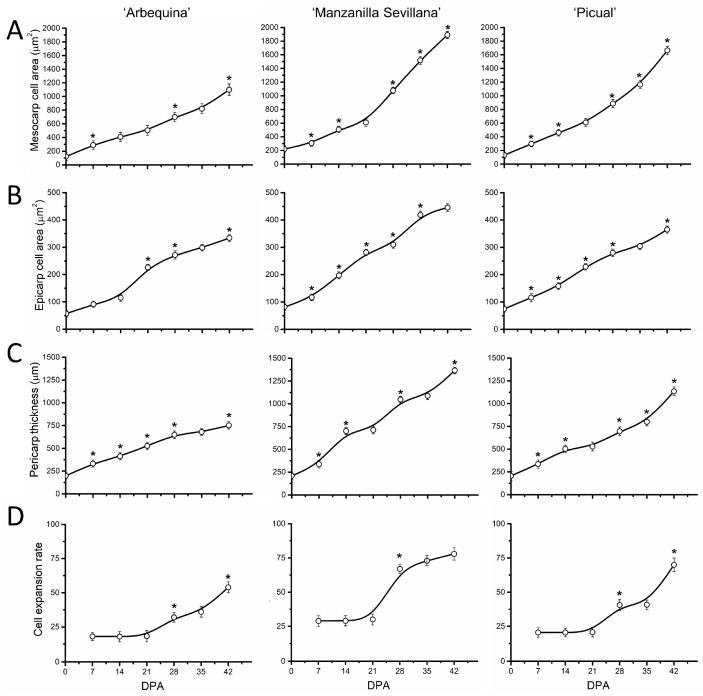
Cell parameters of olive fruit pericarp of ‘Arbequina’, ‘Manzanilla Sevillana’, and ‘Picual’ cultivars during early fruit development. (**A**) Mesocarp cell area (µm^2^), (**B**) epicarp cell area (µm^2^), (**C**) pericarp thickness (µm), and (**D**) cell expansion rate (mesocarp cell area/day) of developing olive fruit at 0, 7, 14, 21, 28, 35, and 42 DPA from the three olive cultivars. The cell area of fruit mesocarp and epicarp cells was measured during early fruit development (staining was with Calcofluor White) using confocal microscopy. Each point is the average of 5 fruits. The values were estimated as means ± SD. Asterisks indicate statistically significant changes based on Tukey’s test (*p* < 0.05) from the preceding point. The differences observed among cultivars are presented in Table S1.

In parallel, to analyze the correlation between fruit size and cell size during early fruit growth, we examined the epicarp and mesocarp cell areas, pericarp thickness, and cell expansion rate during early fruit development (Figure 4). In the three cultivars, the increase in pericarp thickness became detectable from 7 DPA (Figure 4B). Likewise, the area of cells in the mesocarp and epicarp increased by more than 10-fold and 7-fold, respectively, from anthesis to 42 DPA in all three cultivars (Figure 4A,B). However, the mesocarp cell area appeared to be smaller in the ‘Arbequina’ cultivar from 28 to 42 DPA than in ‘Manzanilla Sevillana’ during early fruit growth (Figure 4A, Table S1). The highest cell expansion rates (cell area/day) occurred from 28 to 42 DPA, while pericarp cells expanded at a similar rate for 21 DPA in all three cultivars (Figure 4D).

In addition, different rates of mesocarp cell expansion from 28 to 42 DPA among cultivars led to smaller cell size in ‘Arbequina’ and to larger cell size in ‘Manzanilla Sevillana’ and ‘Picual’ (Figure 4, Table S1). Thus, the smaller cell size in ‘Arbequina’ fruit was due to a lower expansion rate rather than to a shorter period of expansion during early fruit growth.

### 2.3. Late Fruit Development in Olive Cultivars

**Figure 5:**
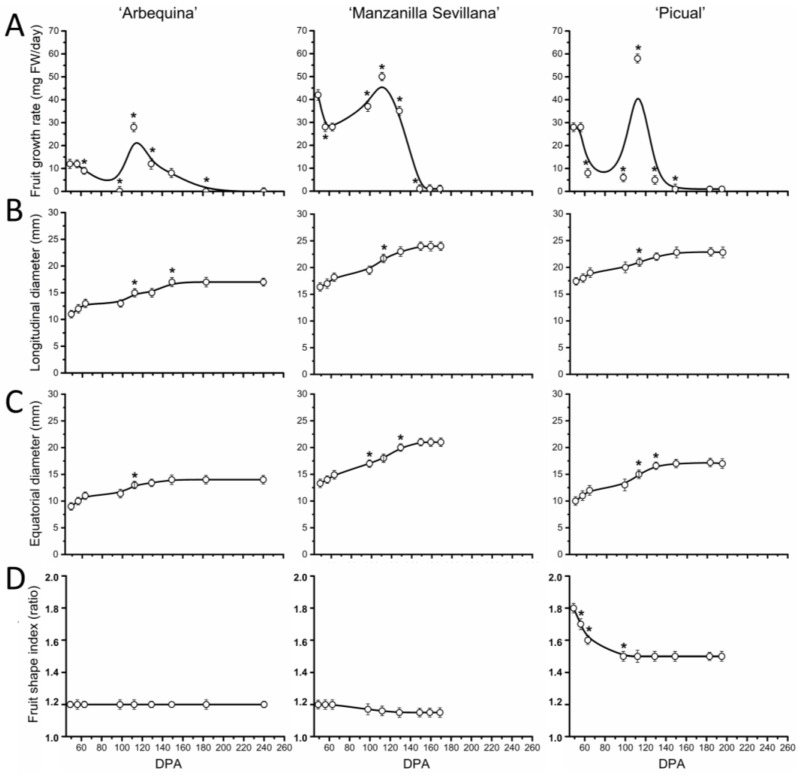
Morphological changes of olive fruit of ‘Arbequina’, ‘Manzanilla Sevillana’, and ‘Picual’ cultivars during fruit growth and ripening. (**A**) Changes in growth rate (mg FW day^−1^), (**B**) longitudinal diameter (mm), (**C**) transverse diameter (mm), and (**D**) fruit shape index of developing olive fruits from the three olive cultivars. Fruit shape index is length-to-width ratio of the fruit. Each point is the average of 5 fruits. The values were estimated as means ± SD. Asterisks indicate statistically significant changes with respect to the preceding point according to Tukey’s test (*p* < 0.05). The differences observed among cultivars are presented in Table S1.

According to the data of fruit growth variables throughout the following fruit growth phase, the three cultivars showed the same patterns of fruit growth and diameters during this phase (Figure 5A–C).

From 56 to 98 DPA, fruit growth rates slowed, and the diameters of olive fruits grew moderately in the three cultivars. At 112 DPA, both parameters rose strongly and then levelled off at the green mature stage (Figure 5). In fact, a secondary increase in fruit weight by 2.5-fold (about 60% of final fruit weight) was evident at the transition between endocarp lignification and green mature stage in the three cultivars (Figure 1). Their fruit growth rate reached its maximum at 112 DPA and then declined until the green mature stage in the three cultivars, but ‘Arbequina’ displayed a slower growth rate than did the other cultivars during this phase (Figure 5, Table S1), consistent with the lower final fruit weight recorded among the three olive cultivars.

**Figure 6:**
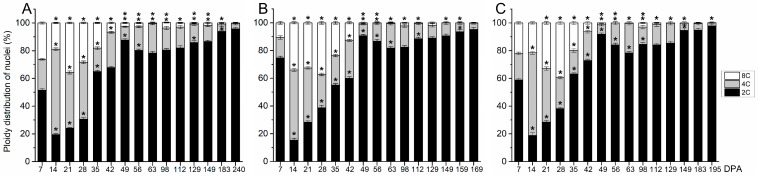
Nuclear ploidy levels from olive fruit pericarps (epicarp and mesocarp) during growth and ripening in (**A**) the ‘Arbequina’, (**B**) ‘Manzanilla Sevillana’, and (**C**) ‘Picual’ olive cultivars. The percentage of nuclei in 2C, 4C, and 8C are shown in the developing fruit pericarp (epicarp and mesocarp). Each point is the average of four samples. Asterisks denote significant differences based on Tukey’s test (*p* < 0.05) from the preceding point, and bars are ±SD The differences observed among cultivars are presented in Table S1.

For a clarification of the contribution of fruit pericarp to the ploidy level of whole fruit and for an evaluation of the ploidy level during late fruit growth in olive, the DNA content of nuclei isolated from olive pericarp was determined using flow cytometric analysis in the three cultivars from 7 DPA to fully ripe stage (Figure 6).

In all cultivars, the maximal proportion of 4C cells was reached at 14 DPA, indicating that intense cell division was activated in the pericarp at this time (Figure 6A–C). The results showed that cell division occurs from 7 to 28 DPA in the fruit pericarps of the three olive cultivars. After 28 DPA, the proportion of 2C cells increased while the proportion of 4C cells decreased in all pericarps up to 49 DPA (endocarp lignification). In addition, a similar proportion of endoreduplicated cells of up to 8C (one endocycle) was detected in pericarp cells between olive cultivars during the early and late phases of olive fruit development (Figure 6, Table S1). Thus, we found variations between early and late fruit growth in the C value proportions observed in olive pericarps, but no difference was detected among cultivars.

**Figure 7:**
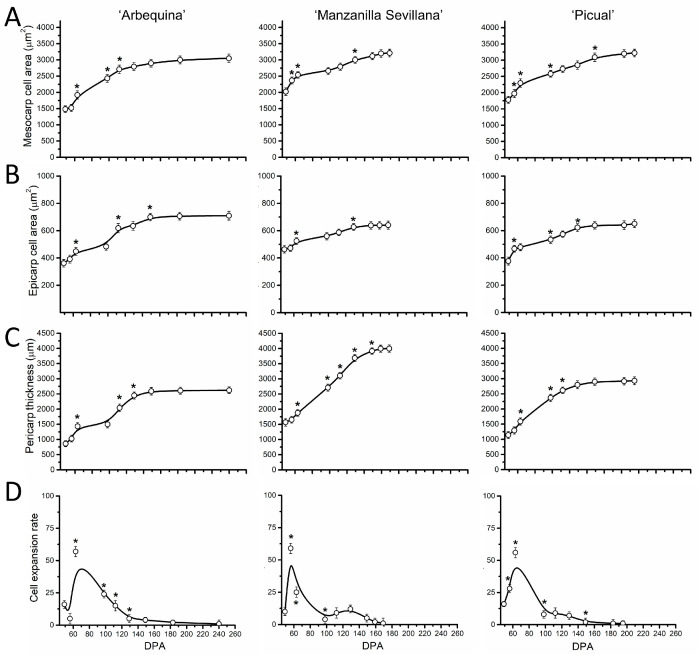
Cell parameters of olive fruit pericarp of ‘Arbequina’, ‘Manzanilla Sevillana’, and ‘Picual’ cultivars during fruit growth and ripening. (**A**) Mesocarp cell area (µm^2^), (**B**) epicarp cell area (µm^2^), and (**C**) pericarp thickness (µm) and (**D**) cell expansion rate (mesocarp cell area/day) of developing olive fruits from the three olive cultivars. Each point is the average of 5 fruits. The values were estimated as means ± SD. Asterisks indicate statistically significant changes based on Tukey’s test (*p* < 0.05) from the preceding point. The differences observed among cultivars are presented in Table S1.

Similar variations occurred for pericarp cell areas and thickness in the three cultivars during late fruit growth (Figure 7A–C). The cell area in the fruit mesocarp at this final stage of olive fruit development did not significantly differ among the three olive cultivars (Figure 7A, Table S1).

In olive fruit, the pericarp thickness increased by more than 2-fold during this phase (Figure 7C). A significant increase in the rate of mesocarp cell expansion was observed between 60 and 80 DPA in the three cultivars (Figure 7D), indicating that intense cell expansion occurs in olive fruit mesocarp after the endocarp lignification.

## 3. Discussion

The present work investigates whether fruit size of large fruited-cultivars could be promoted by an alteration of the cell ploidy profile in contrast to small fruited-cultivars. For this, we measured the ploidy levels and cell size of olive fruit to clarify events that had occurred at the cell level during fruit ontology between large- and small-fruited cultivars.

Here, the data indicated that the basis for fruit size differences between olive cultivars is determined during early fruit growth phase, from anthesis to fruit endocarp lignification (about 50 DPA). All three olive cultivars maintained an initial rapid increase in olive fruit weight over the first 42 days, but differences in fruit weight between large- and small-fruited cultivars were evident as early as 35 DPA and were compounded as fruit developed. During this phase, the gain in the fruit weight rate lasted 10 days longer, on average, in the large-fruited cultivars than in the small-fruited cultivar; thus, the fruit of small-fruited cultivar was the first to reach the complete endocarp lignification. Consequently, the fruit weight of the small-fruited cultivar was augmented by approximately half that of the large-fruited cultivars during this phase. Examining developing fruits from three different olive cultivars by employing flow cytometry and confocal fluorescence microscopic image analyses, we found that cell division in the whole fruit was limited to a short period of early fruit development (21 DPA), coexisting with cell expansion at similar rates in the three cultivars. In addition, the difference in olive fruit shape appeared only after 21 DPA and was accompanied by a halt in cell division and a dramatic burst in cell expansion rates of the fruit mesocarps. Olive fruit of the large-fruited cultivars enlarged, apparently due to relatively higher cell division rates compared with the small-fruited cultivar, whereas the duration of cell division was found to be similar between cultivars.

Moreover, the fruit pericarp (epicarp and mesocarp) showed growth by cell division, even though for a longer duration (28 DPA) than the whole fruit in any of the olive cultivars. These data suggest that the number of cells remains almost constant in the fruit endocarp rather than in the fruit pericarp, and the subsequent fruit growth is due exclusively to cell expansion in all fruit tissues of the olive cultivars. Commonly, tissues closest to the ovules stop dividing earlier than other tissues [33]. In the present study, division ceased earlier in the endocarp than in the pericarp (epicarp and mesocarp), restricting active cell division within the pericarp to an initial period of 3–4 weeks after fertilization, suggesting that the cell number of olive fruit is determined from 28 DPA in all three olive cultivars. The same findings have previously been reported in olive cultivars. However, differences in the extent of cell division have been previously reported for some olive cultivars [23], but a similar period of cell division was found for all olive cultivars in the present study. Thus, the present data indicate spatially and temporally complex regulation of cell division in growing olive fruit, supporting the conclusion that a smaller size of ‘Arbequina’ fruit is explained by smaller cell number of the fruit pericarp due to a lower cell division rate rather than a shorter period of division.

Cell expansion starts directly after fruit set in the mesocarp cells, continuing until ripening, and this is responsible for a rapid and major increase in fruit size [5]. Recently, previous data from RNA-seq indicated the expression of specific genes in the expanding olive fruit [22]. In the present study, all three olive cultivars started cell expansion in a very few days after olive fruit set, concomitantly with cell division, whereas dramatic increases in the expansion rate of olive fruit followed with the cessation of cell division (28 DPA) until endocarp lignification (50 DPA). Although cell expansion continued until the onset of ripening (150 DPA), the process reached its second peak at about 60–80 DPA. However, lower rates of increase in cell area of mesocarp from 28 to 42 DPA in ‘Arbequina’ fruit led to a smaller cell size at 42 DPA, whereas the expansion rates of mesocarp cells proved similar between olive cultivars during late fruit growth. Indeed, greater cell size in large-fruited cultivars was noted compared with small-fruited cultivars during the early stages of fruit growth. In contrast to previous observations in olive fruit [27,29], the present results indicated that the lower fruit rate and smaller size of ‘Arbequina’ fruits compared with ‘Manzanilla Sevillana’ and ‘Picual’ fruits resulted from the slowdown of cell expansion during early fruit development. At 42 DPA, the mesocarp cell area in the small-fruited cultivar ‘Arbequina’ was approximately half that of the large-fruited cultivars ‘Manzanilla Sevillana’ and ‘Picual’. Consequently, the ‘Arbequina’ fruit had half the weight increase of the two larger fruits during this phase. Thus, the smaller size of ‘Arbequina’ fruit was due to reduced cell division activity, but cell size also contributed. A previous study evaluating ‘Arbequina’ fruit developing during drought concluded that carbon and water processes can explain fruit growth, with importance placed on the combination of cell division and expansion [30]. Many previous studies of various fruit-bearing species have supported this suggestion [34,35,36,37,38]. Similarly, we found that olive fruit of the large-fruited cultivars enlarged due to relatively higher cell division and expansion rates compared with the small-fruited cultivar during early fruit development.

The significance of plant endoreduplication in various aspects of cell differentiation, including cell size and growth rate, and in response to environmental stress has previously been described [10,11,39,40,41,42]. In particular, in tomato fruit, the final cell size within the fruit pericarp has been correlated with the level of endoreduplication, with most of the fruit cells displaying highly endoreduplicated nuclei [8]. In olive fruit, a previous study reported that the endoreduplicated cells (8C) reached 15% in whole fruit cell nuclei of the ‘Picual’ cultivar during early fruit growth [22]. Here, we confirm these data and lend complementary support to the hypothesis that endoreduplication occurs in olive pericarp during fruit development in different olive cultivars. In whole fruit, the present cytofluorimetric analysis highlights that the number of cells undergoing endoreduplication (8C) was lower during early fruit growth in ‘Arbequina’ fruit than in ‘Manzanilla Sevillana’ and ‘Picual’ fruits, in agreement with the smaller size reached by ‘Arbequina’ fruit. By contrast, in the pericarps, our analysis showed that, during early fruit growth, the composition of cell populations of pericarps (epicarp and mesocarp) was similar in terms of DNA content between the three cultivars, although the mesocarp cell area in ‘Manzanilla Sevillana’ and ‘Picual’ fruits had already significantly reached higher values than in ‘Arbequina’ fruits. In fact, at 14 DPA, in the three cultivars, fruit pericarp cells with a DNA content of 4C were the most represented, accounting for 53.2–56%, while about 31% of cells had a DNA content of 2C. The high proportion of 4C cells indicated that the pericarp cells of all cultivars were undergoing division (G2/M phase), and cells that had undergone one cycle of endoreduplication were present. At this stage, events of endoreduplication were reflected in a similar DNA content of 8C in olive pericarps of the three cultivars, clearly representing a low degree of endopolyploidization. By contrast, at complete endocarp lignification, pericarp cells with a DNA content of 2C reached a proportion of 98% in the three cultivars. Next, during late fruit growth, pericarp cells collected from the three cultivars with a DNA content of ≥8C were barely detected. Thus, it is worth noting that the cytofluorimetric profiles of olive fruit pericarps differed between early and late olive fruit growth but was similar between the large- and small-fruited cultivars. Therefore, due to the similar number of endoreduplicated cells observed in olive pericarps among cultivars, exclusively during early fruit growth, our data suggest that endoreduplication may be not associated with the higher weight registered in large-fruited cultivars.

## 4. Materials and Methods

### 4.1. Plant Material and Cytological Analysis

Three cultivars of olive (*Olea europaea* L. subsp. *europaea* var. *europaea*) differing markedly in fruit size, i.e., ‘Arbequina’, ‘Picual’, and ‘Manzanilla Sevillana’, were used (Figure 1). Olive trees of the three cultivars were grown under drip irrigation and fertirrigation in an orchard near Badajoz (Spain). From these trees (10 trees/cultivar), flowers were collected at the anthesis stage (0 days post-anthesis, DPA) and fruits were sampled at 7, 14, 21, 28, 35, 42, and 49 DPA, spanning a time from the fruit set to the time of endocarp lignification [22,43,44,45]. In addition, after endocarp lignification, fruits of the three olive cultivars at specific developmental stages were randomly tagged, collected, and processed as previously described [46,47,48]. A total of 500 fruits from 10 olive trees per cultivar were used for each developmental stage. To minimize the effects related to asynchronous fruits ripening within the same tree, we picked fruits with similar pigmentation from all around the external parts of the tree canopy. Flowers at the anthesis stage and whole fruits of ‘Arbequina’, ‘Picual’, and ‘Manzanilla Sevillana’ olive cultivars were weighed, and the longitudinal and transverse diameters were measured at different developmental stages [22,48]. Flowers at the anthesis stage and whole fruit as well as fruit pericarp samples at different developmental stages were collected for cytological and ploidy analyses.

### 4.2. Cytological Analysis

The cytological study was performed as described by [22]. At least three biological replicates were made at each stage. The cell size (cell area) and pericarp thickness (cross-section) were determined using a CellProfiler image analysis system [49].

### 4.3. Flow Cytometry Analysis

The cell division period was precisely determined using flow cytometry with nucleus ploidy profiles taken from ovaries and fruits [22,30,50]. The DNA content (C value) of the olive fruit cells was assessed using flow cytometry with the use of internal calibration standards [22,30,50]. The C values were determined following [51]: 2C DNA (pg) = (mean of the problem sample G1 peak × 2C DNA content of the standard [pg])/mean of the standard G1 peak.

### 4.4. Statistical Analysis

All the experiments were carried out in triplicate, and all data are presented as mean ± standard deviation. Variables of three replicates were compared using Tukey’s multiple-range test, and a *p* value of 0.05 was considered significant. The differences observed among cultivars were assessed by one-way ANOVA followed by post-hoc (Bonferroni) test (Table S1).

## 5. Conclusions

We conclude that the basis for fruit size differences between olive cultivars is determined mainly in the early fruit growth phase. Although fruit pericarp cells increased in ploidy level during early fruit development, the cells displayed a low degree of endopolyploidization, and no association between ploidy level and size in cells was found in the pericarps. Thus, endoreduplication does not appear to be relevant to olive fruit size or to eventual fruit development. The present study indicates that the olive fruit of large-fruited cultivars must have acquired more active cell division and expansion, emphasizing the value of early growth events in determining the final fruit size in olive.

## Data Availability

Data is contained within the article or Supplementary Material.

## Supplementary Materials

The following supporting information can be downloaded at: https://www.mdpi.com/article/10.3390/plants13070990/s1, Table S1. Comparisons among olive cultivars for the values at each stage of fruit development. Differences between a small-fruited cultivar, ‘Arbequina’, and two large-fruited cultivars, ‘Picual’ and ‘Manzanilla Sevillana’, were assessed by one-way ANOVA followed by post-hoc (Bonferroni) test. *, *p* ≤ 0.05; **, *p* < 0.01.
