# Supplementary material for: Variations in Fruit Ploidy Level and Cell Size between Small- and Large-Fruited Olive Cultivars during Fruit Ontogeny
Source: Plants (Basel). 2024 Mar 29;13(7):990. doi: 10.3390/plants13070990 (PMC11013306; doi:10.3390/plants13070990)
Supplement: Supplementary file 1 [file plants-13-00990-s001.zip › plants-2892161-supplementary.pdf]

SUPPLEMENTARY DATA

Table S1. Comparisons among olive cultivars for the values at each stage (days post-anthesis, DPA) of fruit development. Differences between a small-fruited cultivar, 'Arbequina', and two large-fruited cultivars, 'Picual' and 'Manzanilla Sevillana', were assessed by one-way ANOVA followed by post-hoc (Bonferroni) test. *, $p \leq 0.05$; **, $p < 0.01$.

Figure 1

DPA	ANOVA	Arbequina vs Picual	Arbequina vs Manzanilla Sevillana
0			
7			
14			
21			
28			
35	**	**	**
42	**	**	**
49	**	**	**
56	**	**	**
63	**	**	**
98	**	**	**
112	**	**	**
129	**	**	**
149	**	**	**
V	**	**	**
FR	**	**	**

Figure 2A

DPA	ANOVA	Arbequina vs Picual	Arbequina vs Manzanilla Sevillana
7			
14			
21			
28	**		**
35	**	**	**
42	**	**	**
49	**	**	**

Figure 3

DPA	ANOVA (2C)	Arbequina vs Picual	Arbequina vs Manzanilla Sevillana
-7			
0	**		**
7	**	*	**
14	**	**	**
21			
28	**	*	**
35			
42			

DPA	ANOVA (4C)	Arbequina vs Picual	Arbequina vs Manzanilla Sevillana
-7			
0	**		**
7	**		**
14	**	**	**
21	*	*	
28			
35			
42			

DPA	ANOVA (8C)	Arbequina vs Picual	Arbequina vs Manzanilla Sevillana
-7			
0			
7	*		*
14	*	*	*
21	*	*	*
28	**	**	**
35	*	*	*
42			

Figure 4A

DPA	ANOVA	Arbequina vs Picual	Arbequina vs Manzanilla Sevillana
0			
7			
14			
21			
28	*		*
35	**	*	**
42	*		*
49	**		**

Figure 5A

DPA	ANOVA	Arbequina vs Picual	Arbequina vs Manzanilla
56	**	**	**
63	**		**
98	**		**
112	**	**	**
129	**	**	**
149	**	**	**
V			
FR			

Figure 6

DPA	ANOVA (8C)	Arbequina vs Picual	Arbequina vs Manzanilla
7			
14			
21			

28
35
42
49
56
63
98
112
129
149
V
FR

Figure 7A

DPA	ANOVA	Arbequina vs Picual	Arbequina vs Manzanilla
56	*		*
63			
98			
112			
129			
149			
V			
FR			
